# Protein Intake and Frailty in Older Adults: A Systematic Review and Meta-Analysis of Observational Studies

**DOI:** 10.3390/nu14132767

**Published:** 2022-07-05

**Authors:** Hélio José Coelho-Junior, Riccardo Calvani, Anna Picca, Matteo Tosato, Francesco Landi, Emanuele Marzetti

**Affiliations:** 1 Department of Geriatrics and Orthopedics, Università Cattolica del Sacro Cuore, L.go F. Vito 1, 00168 Rome, Italy; francesco.landi@unicatt.it (F.L.); emanuele.marzetti@policlinicogemelli.it (E.M.); 2 Fondazione Policlinico Universitario “Agostino Gemelli” IRCCS, L.go A. Gemelli 8, 00168 Rome, Italy; anna.picca@policlinicogemelli.it (A.P.); matteo.tosato@policlinicogemelli.it (M.T.)

**Keywords:** anorexia, physical function, walking speed, muscle strength, dynapenia, nutrition, elderly, diet

## Abstract

Background: The present systematic review and meta-analysis investigated the cross-sectional and longitudinal associations between protein intake and frailty in older adults. Methods: We conducted a systematic review and meta-analysis of cross-sectional and longitudinal studies that investigated the association between protein intake and frailty in older adults. Cross-sectional, case-control, and longitudinal cohort studies that investigated the association between protein intake and frailty as a primary or secondary outcome in people aged 60+ years were included. Studies published in languages other than English, Italian, Portuguese, or Spanish were excluded. Studies were retrieved on 31 January 2022. Results: Twelve cross-sectional and five longitudinal studies that investigated 46,469 community-dwelling older adults were included. The meta-analysis indicated that absolute, bodyweight-adjusted, and percentage of protein relative to total energy consumption were not cross-sectionally associated with frailty. However, frail older adults consumed significantly less animal-derived protein than robust people. Finally, high protein consumption was associated with a significantly lower risk of frailty. Conclusions: Our pooled analysis indicates that protein intake, whether absolute, adjusted, or relative to total energy intake, is not significantly associated with frailty in older adults. However, we observed that frail older adults consumed significantly less animal protein than their robust counterparts.

## 1. Introduction

Frailty is a state of multisystem derangement and poor psychosocial support [1,2]. The prevalence of frailty increases with age and is highest among those hospitalized or institutionalized [3,4]. Frailty progression increases the vulnerability to many negative events, including falls and fractures, disability, hospitalization, nursing home placement, and death [5,6,7]. Such a scenario requires a massive utilization of healthcare services, making frailty a costly condition [8]. As such, frailty is recognized as a major public health problem [1,2].

Inadequate nutritional habits are an important modifiable risk factor for frailty [9,10,11]. Particularly, numerous observational studies have observed that a high protein intake is negatively associated with the presence of frailty in older adults [12,13,14]. These findings were supported by a systematic review and meta-analysis published in 2018 [15]. However, since then, other investigations have been published confirming or rejecting those results [16,17]. Furthermore, no conclusions were drawn on longitudinal associations between protein intake and frailty [15].

Based on these premises, the present study aimed to update and extend prior results by conducting a robust search strategy in multiple databases and different languages to recover as much information as possible on the cross-sectional and longitudinal association between protein intake and frailty in older adults.

## 2. Materials and Methods

This is a systematic review and meta-analysis of observational studies that investigated cross-sectional and longitudinal associations between protein intake and frailty. The study was fully performed by investigators, and no librarian was part of the team. The study is compliant with the guidelines of the Meta-analysis of Observational Studies in Epidemiology (MOOSE) [18] and the Cochrane Handbook for Systematic Reviews and Interventions [19]. An a priori protocol was established and registered on PROSPERO, which is an international prospective register of systematic reviews [CRD42020165762].

### 2.1. Eligibility Criteria

Inclusion criteria were: (1) observational studies (e.g., case-control, cross-sectional, and cohort longitudinal studies) that investigated the association between protein intake and frailty; (2) participants aged 60 years or older; (3) frailty identified using a validated tool; and (4) published studies in English, Italian, Portuguese, or Spanish languages. To be included in the meta-analysis of cross-sectional studies, investigations should provide the mean and standard deviation (SD) of case (i.e., high protein intake [HPI]) and control groups (i.e., low protein intake, LPI) or at least two groups divided according to protein consumption, and the sample size of each group, or Pearson’s correlation coefficient (r)/betas (β)/odds ratio (OR) values for the association between protein intake and frailty. For the meta-analysis of longitudinal studies, investigations should provide the number of participants, β, OR, hazard ratio (HR), and/or the risk ratio (RR) for the development of frailty according to protein consumption levels. We excluded randomized controlled trials, quasi-experimental, cross-over, and preclinical studies, and any investigations that examined the effects of nutritional interventions alone or combined with other interventions (e.g., physical exercise) on frailty. Studies that enrolled participants with gastrointestinal and/or renal diseases, anorexia, cancer, or any condition that may directly impair protein metabolism (e.g., maple syrup urine disease and tyrosinemia) were also excluded.

### 2.2. Search Strategy and Selection Criteria

Studies published on or before 31 January 2022 were retrieved from the following six electronic databases by one investigator: (1) MEDLINE (PubMed interface); (2) SCOPUS (Elsevier interface); (3) EMBASE (OVID interface), (4) CINAHL (EBSCO interface); (5) AgeLine (EBSCO interface); and (6) Food Science Source (EBSCO interface). Further eligible articles were identified by checking the reference lists of the retrieved articles. In addition, citation searches on key articles were performed in Google Scholar and ResearchGate. Initially, a search strategy was designed using keywords, MeSH terms, and free text words (e.g., protein intake, frailty, older adults). Afterwards, keywords and subject headings were exhaustively combined using Boolean operators. The complete search strategy is shown in Supplementary Material S1.

### 2.3. Data extraction, Quality Assessment, and Risk of Bias

The titles and abstracts of the retrieved articles were screened for eligibility by two researchers (HJCJ and RC). The full text was consulted if the abstract did not provide enough information for final evaluation. Two reviewers (HJCJ and RC) extracted the coded variables (i.e., methodological quality, risk of bias, and characteristics of the studies) using a standardized coding form. A third researcher was consulted to solve disagreements (EM), if necessary. The quality of reporting for each study was performed by two researchers (HJCJ and RC) using the Quality Assessment Tool for Observational Cohort and Cross-Sectional Studies of the National Institute of Health [20]. This tool contains 14 questions that assess several aspects that are associated with the risk of bias, type I and type II errors, transparency, and confounding factors. Studies were positive for item 8 if they investigated protein sources and/or distribution. Items 6, 7, and 13 do not refer to cross-sectional studies and were removed from the quality analysis. The maximum scores for cross-sectional and longitudinal studies were 11 and 14, respectively. The agreement rate for quality assessment between reviewers was 98%.

### 2.4. Statistical Analysis

Meta-analysis was conducted using Revman 5.4.1 (Cochrane Collaboration, Copenhagen, Denmark) and STATA 13 (StataCorp, College Station, TX, USA). Effect sizes (ESs) were measured using: (1) means and SDs and (2) logOR and confidence intervals (CIs). Central and dispersion values were obtained from included studies or calculated according to Cochrane guidelines [19]. Specifically, medians were assumed as means when studies reported symmetrical data. SDs were calculated from CIs and standard errors (SEs) according to the following formulas:

SD1 = √N × (Upper limit − Lower limit)/3.92

SD2 = SE × √N

From interquartile range (IQR), SDs were obtained according to the formulas proposed by Luo [21] and Shi [22]. OR was calculated using the number of participants allocated into the HPI and LPI groups or obtained from β values. Results were log-transformed (base 10) before being analyzed. A single pairwise comparison was created when multiple studies referred to the same database using the formulas proposed by the Cochrane group [19]. Pooled ES was calculated based on standard mean differences (SMDs) and logOR. Due to the variability of sample characteristics, a random-effect model was used to calculate the pooled ES.

## 3. Results

### 3.1. Literature Search

**Figure 1:**
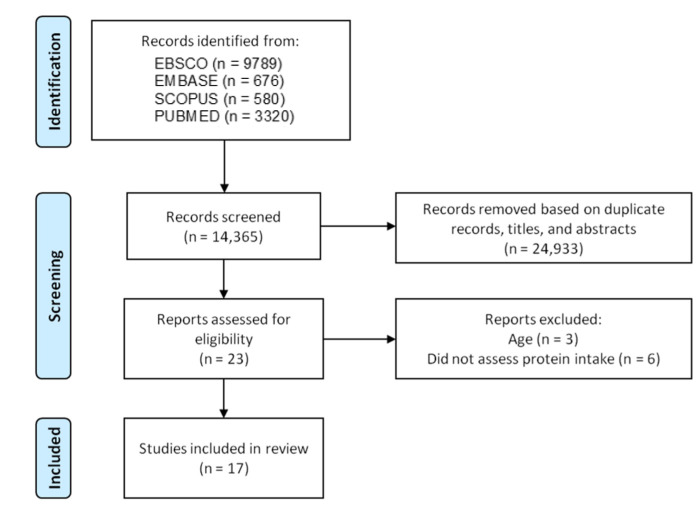
Study flowchart.

Figure 1 depicts the study flowchart. An amount of 14,365 entries were retrieved from electronic databases and hand searches. Of these, 14,342 were excluded based on duplicate data, titles, or abstracts. Twenty-three studies were fully reviewed and assessed for eligibility. Six articles were excluded (Supplementary Material S2), and seventeen investigations were included in the study.

### 3.2. Main Characteristics of the Included Studies

The main characteristics of the included cross-sectional studies are shown in Table 1. Twelve cross-sectional studies [12,13,14,16,17,23,24,25,26,27,28,29] that examined 13,593 community-dwelling older adults with a mean age of approximately 73.0 years were included. In all studies, frailty was identified according to the frailty phenotype (FP) [30]. One study [28] used both the frailty phenotype and the Kihon checklist (KCL) [31], while another study [16] used three instruments: FP, FRAIL scale [32], and the study of osteoporotic fracture (SOF) instrument [33]. Most studies assessed dietary habits using 24 h dietary recalls. Self-administered diet history questionnaires were used in four studies, and food frequency questionaries (FFQs) were used in three studies.

The main characteristics of the included longitudinal studies are shown in Table 2. Five longitudinal studies [34,35,36,37,38] that investigated 32,876 community-dwelling older adults with a mean age at baseline of approximately 69.4 years were included. The mean follow-up period was 3.2 years (ranging from 2–4.6 years). Four studies identified participants with frailty using FP, while one study [35] applied a model of social frailty [39]. Nutritional habits were recorded using FFQs, 3-day food records, and diet history. 

### 3.3. Quality Assessment

The quality assessment of the cross-sectional and longitudinal studies is shown in Supplementary Material S3. The overall score of cross-sectional studies ranged from 6 to 8. All studies clearly stated the research question (item 1), specified the study population (item 2), recruited participants from the same or a similar population (item 4), clearly defined and used valid and reliable exposure (item 9), and the outcome variables (item 11). Six studies investigated different levels of exposure (item 8), two investigations did not adjust their results according to confounding parameters (item 14), and one study did not report if the participation rate of eligible persons was of at least 50% (item 3). None of the studies justified the sample size (item 5), assessed the exposure more than once, or reported if investigators were blinded to the exposure of the participants (item 12).

The overall score of longitudinal studies ranged from 8 to 10. All studies established the research question (item 1), specified the study population (item 2), recruited participants from the same or a similar population (item 4), measured the exposure of interest before the outcome was measured (item 6), used a timeframe sufficient enough to expect an association between exposure and outcome (item 7), clearly defined and used valid and reliable exposure (item 9), and the outcome (item 11) measures, and adjusted their results according to confounding parameters (item 14). Four studies investigated a study population with a participation rate of eligible persons of at least 50% (item 3), two studies investigated different levels of exposure (item 8), and one investigation reported a loss of follow-up after a baseline of 20% or less (item 13). No studies assessed the exposure more than once (item 10).

### 3.4. Cross-Sectional Associations between Protein Intake and Prefrailty

**Figure 2:**
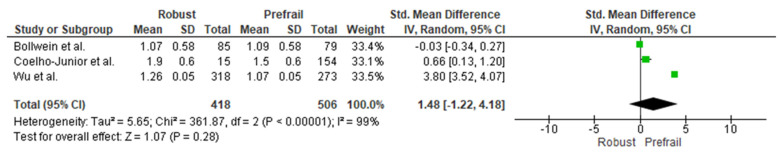
Mean and standard deviation for protein intake in robust and prefrail people [16,24,27].

Figure 2 shows the differences in protein intake between prefrail and robust older adults. The pooled analysis indicated that there were no significant differences between groups (SMD = 1.48, 95%CI: −1.22–4.18, *p* = 0.28).

### 3.5. Cross-Sectional Associations between Protein Intake and Frailty Using Continuous Data

**Figure 3:**
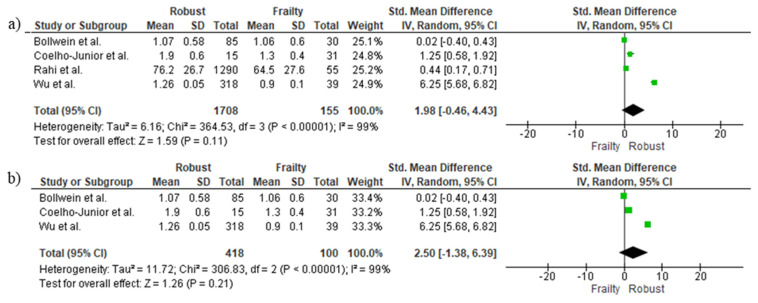
Mean and standard deviation for protein intake in robust and frail people. (**a**) All studies, (**b**) Only studies reporting protein intake adjusted by body weight [14,16,24,27].

Figure 3 shows the differences in protein intake between frail and robust older adults. The pooled analysis indicated that there were no significant differences between groups (SMD = 1.98, 95%CI: −0.46–4.43, *p* = 0.11; Figure 3a). Results remained non significant when only studies reporting protein intake adjusted by body weight (BW) were analyzed (SMD = 2.50, 95%CI: −1.38–6.39, *p* = 0.21; Figure 3b).

### 3.6. Cross-Sectional Associations between Protein Intake and Frailty Using Binary Data

**Figure 4:**
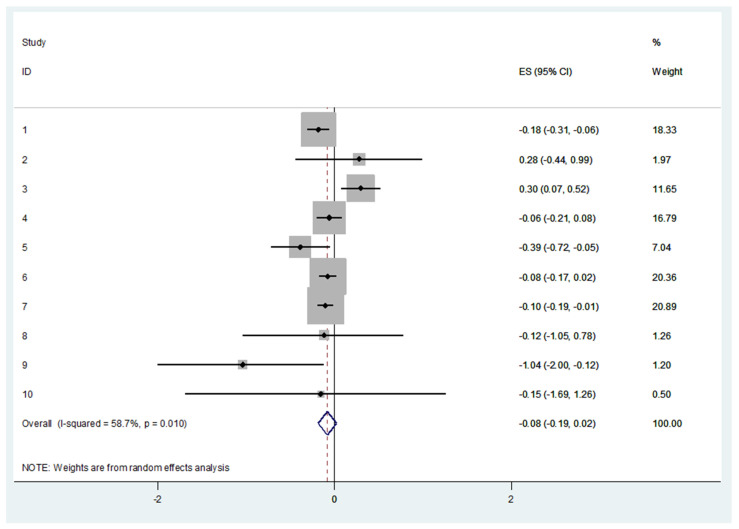
Log10 for the cross-sectional associations between protein intake and frailty using binary data.

Figure 4 shows the pooled analysis of the 10 studies that investigated the association between protein intake and frailty in older adults. The association was not significant (log10 = −0.082, 95%CI = −0.187–0.023, *p* = 0.127). Data remained non significant when the analyses were conducted according to protein consumption levels (absolute, adjusted to BW, and percentage of protein relative to total energy consumption).

### 3.7. Cross-Sectional Differences in Protein Sources between Frail and Robust People

**Figure 5:**
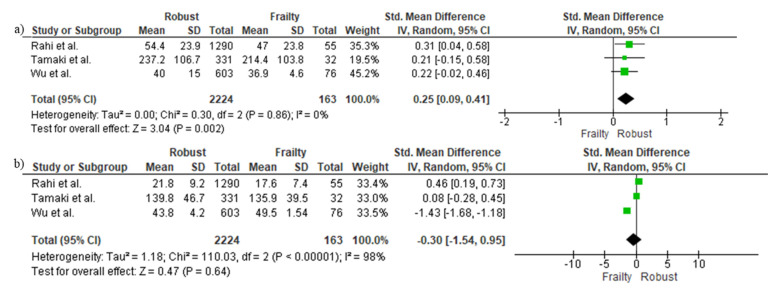
Mean and standard deviation for (**a**) animal- and (**b**) plant-based protein intake in robust and frail people [14,27,28].

Figure 5 shows the pooled analysis of three studies that investigated the association between protein sources and frailty in older adults. Results indicated that frail older adults consumed significantly less animal-derived protein (SMD = 0.25, 95%CI= 0.09–0.41, *p* = 0.002; Figure 5a), but not plant-based protein (SMD = −0.30, 95%CI = −1.54–0.95, *p* = 0.64; Figure 5b) when compared to robust people.

### 3.8. Longitudinal Associations between Protein Intake and Incidence of Frailty

**Figure 6:**
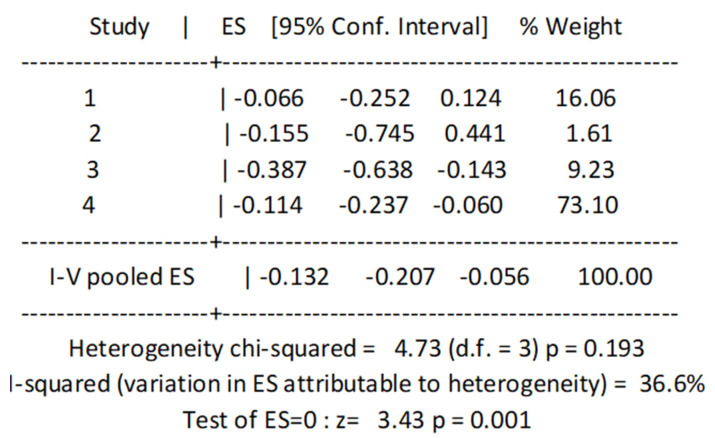
Log10 for the longitudinal associations between protein intake and incidence of frailty. ES, Effect size.

Figure 6 shows the pooled analysis of the four studies that investigated the longitudinal association between protein intake and frailty in older adults. High protein consumption was associated with a significantly lower risk of frailty (log10 = −0.132, 95%CI = −0.207–0.056, *p* = 0.001).

## 4. Discussion

The present study examined more than 45,000 community-dwelling older adults to investigate the association between protein intake and frailty. No significant differences in protein consumption were observed between prefrail and frail older adults relative to robust peers. These findings were supported by the analysis of binary data, which indicated that protein intake, whether absolute, adjusted, or relative to total energy intake, was cross-sectionally associated with frailty. However, frail older adults consumed significantly less animal-based protein than their robust counterparts. Furthermore, the pooled analysis of longitudinal studies indicated that higher protein consumption was associated with a lower risk of incident frailty.

The current findings are in contrast with those of a prior systematic review and meta-analysis, which reported a significant negative association between protein intake and the prevalence of frailty in older adults [15]. A possible explanation for this discrepancy might be that, in our previous study [15], results were not stratified according to protein sources. Indeed, findings of the present investigation indicate that frail older adults consumed less animal-based protein than robust people.

Sarcopenia is a neuromuscular disease characterized by substantial muscle atrophy, dynapenia, and the loss of physical function [40,41]. This condition shares numerous physiopathological markers and clinical aspects with frailty [42,43,44]; thus, it has been suggested that sarcopenia might be a substratum for frailty development [43]. As such, most of the possible effects of protein intake on frailty are thought to be associated with changes in sarcopenia-related parameters.

Muscle mass is regulated by a dynamic balance between muscle protein synthesis (MPS) and muscle protein breakdown (MPB) [45,46,47,48,49]. An imbalance in age-related protein metabolism toward MPB promotes muscle atrophy, especially in those with a predominance of type II fast-twitch fibers [50,51,52,53]. On the other hand, protein intake is a major regulator of muscle metabolism via the increase in amino acid availability. Hyperaminoacidemia stimulates MPS through the activation of ribosomal protein kinase S6 (S6K1) and 4E-binding protein 1 (4EBP1) under the coordination of the mammalian target of rapamycin (mTOR) [45,46,47,48,49].

Animal and plant proteins evoke different anabolic responses owing to varying digestibility rates and branched-chain amino acid (BCAA) content [54,55]. Digestibility refers to the proportion of amino acids that become available for MPS after digestion and absorption of dietary proteins [55]. Animal-based proteins are characterized by digestibility rates higher than 90%, which instead barely reaches 50% for plant-based proteins [55]. Furthermore, animal foods are recognized as the primary source of high-quality proteins by having a higher content of BCAAs than vegetal proteins [54,56]. These data are important because BCAAs, mainly leucine, greatly stimulate MPS by acting on mTOR and its downstream effectors [57,58,59].

Taken together, it is possible that older adults who consume low amounts of animal protein do not properly stimulate MPS, which in turn may contribute to muscle atrophy, neuromuscular dysfunction, loss of mobility, sarcopenia, and, consequently, frailty.

However, some investigations have observed that a high intake of vegetal proteins was associated with better mobility [60] and a low prevalence of frailty in adults [61]. These findings suggest that an adequate intake of plant-based protein could also properly stimulate MPS [43]. Nevertheless, longitudinal studies did not show differences in incident frailty according to protein sources.

Numerous other protein-related parameters can potentially influence the relationship between dietary protein and frailty and might explain the current results, which were not investigated in the included studies. For instance, Ten Haaf et al. [62] found that a more widely spread protein distribution across main meals was associated with faster walking speed in older adults. Loenneke et al. [63] observed that older adults who consumed two or more meals with 30 g of protein in each were stronger and had more muscle mass compared with those who consumed one or no meals with at least 30 g of protein. In addition, authors observed that MPS is maximally stimulated by the ingestion of 0.4 g of high-quality dietary protein per kg of BW [64]. Therefore, the possibility that one or more of these aspects could have impacted the findings of longitudinal studies cannot be ruled out.

Our study has several limitations that deserve discussion. Firstly, all investigations examined community-dwelling older adults, and so extrapolations to hospitalized people or those living in long-term institutions should be made with caution. Secondly, the results regarding protein sources were based on means and SDs, given the limited number of studies that conducted regression analyses. This indicates that the results were not adjusted for numerous covariables, including physical activity levels [62], oral health [65], and the presence of comorbidities [66]. Particularly, recent findings from the SPRINTT project showed that a multicomponent intervention, which involved a daily protein intake of at least 1.0–1.2 g/kg of BW and a physical activity intervention, reduced the incidence of mobility disability in older adults with physical frailty and sarcopenia [67]. Thirdly, the limited number of included studies did not allow meta-regression, dose-response, risk of bias, or “trim and fill” analyses to be conducted. Fourthly, most studies used FP to identify frailty, which precludes the generalization of findings to older adults in whom frailty is diagnosed according to other tools. Fifthly, substantial heterogeneity was observed in the methods used to assess nutritional habits.

## 5. Conclusions

Our pooled analysis indicates that protein intake, whether absolute, adjusted, or relative to total energy intake, is not significantly associated with frailty in older adults. However, we observed that frail older adults consumed significantly less animal protein than their robust counterparts. No significant differences in frailty status were observed according to the amount of vegetal protein consumed. These findings indicate that protein sources might have a key role in the development of frailty. Furthermore, a higher protein consumption is longitudinally associated with a lower risk of frailty. Further studies using frailty assessment tools other than FP and taking into account protein-related parameters (e.g., ingestion patterns) are required to confirm and expand the current results.

## Figures and Tables

**Table 1:** Main Characteristics of Cross-sectional Studies Included in the Meta-analysis [12,13,14,16,17,23,24,25,26,27,28,29].

Year	Author	Country	Sample Characteristics	Sample Size (n)	Mean (Years)	Protein Intake	Dietary Intake Assessment Method	Frailty Assessment Tool
2006	Bartali et al.	Italy	Community-dwelling older adults	802	74.0	—	Food frequency questionnaire	Frailty phenotype
2013	Bollwein et al.	Germany	Community-dwelling older adults	195	83	76.6 g	Food frequency questionnaire	Frailty phenotype
2017	Castaneda-Gameros et al.	United Kingdom	Community-dwelling women	76	70.5	—	24 h dietary recall	Frailty phenotype
2020	Coelho-Junior et al.	Brazil	Community-dwelling older adults	200	~67.4	~1.6 g/d/kg body weigh	24 h dietary recall	Frailty phenotype, FRAIL scale, SOF
2021	Hayashi et al.	Japan	Community-dwelling older adults	120	73	69.4 g	Food frequency questionnaire	Frailty phenotype
2021	Kaimoto et al.	Japan	Community-dwelling older men	815	74.9	~79.9 g	Self-administered diet history questionnaire	Frailty phenotype
2013	Kobayashi et al.	Japan	Community-dwelling women	481	74.7	74.0 g	Self	Frailty phenotype
2017	Kobayashi et al.	Japan	Community-dwelling women	2108	74	74.0 g	Self	Frailty phenotype
2016	Rahi et al.	France	Community-dwelling women	1345	~75,6	~70.3 g	24 h dietary recall	Frailty phenotype
2013	Smit et al.	USA	Community-dwelling older adults	4731	60+	~66.9 g	24 h dietary recall	Frailty phenotype
2018	Tamaki et al.	Japan	Community-dwelling older adults	800	72.6	—	Self-administered diet history questionnaire	KCL and frailty phenotype
2021	Wu et al.	Taiwan	Community-dwelling older adults	1920	~74	—	24 h dietary recall	Frailty phenotype

**Table 2:** Main Characteristics of Longitudinal Studies Included in the Meta-analysis [34,35,36,37,38].

Year	Author	Follow-Up Period (Years)	Country	Sample Characteristics	Sample Size (n)	Mean Age (Years)	Protein Intake	Dietary Intake Assessment Method	Frailty
2010	Beasley et al.	3.0	USA	Community-dwelling older adults	24417	65–79	~1.1 g/d/kg body weight	Food frequency questionnaire	Frailty phenotype
2020	Huang et al.	3.0	Japan	Community-dwelling older adults	429	69.4	1.1 g/d/kg body weight	Food frequency questionnaire	Social frailty
2019	Otsuka et al.	2.0	Japan	Community-dwelling women	283	~72	~77.2 g	3-day food record	Frailty phenotype
2016	Sandoval-Insausti et al.	3.5	Spain	Community-dwelling older adults	1822	68.7	—	Diet history	Frailty phenotype
2014	Shikany et al.	4.6	USA	Community-dwelling older men	5925	65+	—	Food frequency questionnaire	Frailty phenotype

## Data Availability

Data are available in the manuscript.

## Supplementary Materials

The following supporting information can be downloaded at: https://www.mdpi.com/article/10.3390/nu14132767/s1, Supplementary material S1, the complete search strategy; Supplementary material S2, Six articles which were excluded; Supplementary material S3, Quality analysis.

## References

[1] Dent E., Martin F.C., Bergman H., Woo J., Romero-Ortuno R., Walston J.D. (2019). Management of Frailty: Opportunities, Challenges, and Future Directions. Lancet.

[2] Hoogendijk E.O., Afilalo J., Ensrud K.E., Kowal P., Onder G., Fried L.P. (2019). Frailty: Implications for Clinical Practice and Public Health. Lancet.

[3] Coelho-Júnior H.J., Marzetti E., Picca A., Calvani R., Cesari M., Uchida M. (2020). Prevalence of Prefrailty and Frailty in South America: A Systematic Review of Observational Studies. J. Frailty Aging.

[4] Ofori-Asenso R., Chin K.L., Mazidi M., Zomer E., Ilomaki J., Zullo A.R., Gasevic D., Ademi Z., Korhonen M.J., LoGiudice D. (2019). Global Incidence of Frailty and Prefrailty Among Community-Dwelling Older Adults. JAMA Netw. Open.

[5] Kojima G. (2016). Frailty as a Predictor of Hospitalisation among Community-Dwelling Older People: A Systematic Review and Meta-Analysis. J. Epidemiol. Community Health.

[6] Kojima G. (2017). Frailty Significantly Increases the Risk of Fractures among Middle-Aged and Older People. Evid. Based Nurs..

[7] Vermeiren S., Vella-Azzopardi R., Beckwée D., Habbig A.-K., Scafoglieri A., Jansen B., Bautmans I., Bautmans I., Verté D., Beyer I. (2016). Frailty and the Prediction of Negative Health Outcomes: A Meta-Analysis. J. Am. Med. Dir. Assoc..

[8] Hajek A., Bock J.-O., Saum K.-U., Matschinger H., Brenner H., Holleczek B., Haefeli W.E., Heider D., König H.-H. (2018). Frailty and Healthcare Costs-Longitudinal Results of a Prospective Cohort Study. Age Ageing.

[9] Lorenzo-López L., Maseda A., de Labra C., Regueiro-Folgueira L., Rodríguez-Villamil J.L., Millán-Calenti J.C. (2017). Nutritional Determinants of Frailty in Older Adults: A Systematic Review. BMC Geriatr..

[10] Ward R.E., Orkaby A.R., Chen J., Hshieh T.T., Driver J.A., Gaziano J.M., Djousse L. (2019). Association between Diet Quality and Frailty Prevalence in the Physicians’ Health Study. J. Am. Geriatr. Soc..

[11] Coelho-Junior H.J., Marzetti E., Picca A., Cesari M., Uchida M.C., Calvani R. (2020). Protein Intake and Frailty: A Matter of Quantity, Quality, and Timing. Nutrients.

[12] Kobayashi S., Asakura K., Suga H., Sasaki S. (2013). Three-generation Study of Women on Diets and Health Study Group High Protein Intake Is Associated with Low Prevalence of Frailty among Old Japanese Women: A Multicenter Cross-Sectional Study. Nutr. J..

[13] Kaimoto K., Yamashita M., Suzuki T., Makizako H., Koriyama C., Kubozono T., Takenaka T., Ohishi M., Kanouchi H. (2021). Association of protein and magnesium intake with prevalence of prefrailty and frailty in community-dwelling older Japanese women. J. Nutr. Sci. Vitaminol..

[14] Rahi B., Colombet Z., Gonzalez-Colaço Harmand M., Dartigues J.F., Boirie Y., Letenneur L., Feart C. (2016). Higher Protein but Not Energy Intake Is Associated With a Lower Prevalence of Frailty Among Community-Dwelling Older Adults in the French Three-City Cohort. J. Am. Med. Dir. Assoc..

[15] Coelho-Júnior H.J., Rodrigues B., Uchida M., Marzetti E. (2018). Low Protein Intake Is Associated with Frailty in Older Adults: A Systematic Review and Meta-Analysis of Observational Studies. Nutrients.

[16] Coelho-Júnior H.J., Calvani R., Picca A., Gonçalves I.O., Landi F., Bernabei R., Cesari M., Uchida M.C., Marzetti E. (2020). Protein-Related Dietary Parameters and Frailty Status in Older Community-Dwellers across Different Frailty Instruments. Nutrients.

[17] Hayashi T., Fukuda Y., Sato R., Ogasawara M., Tamura K. (2021). Association of Physical Prefrailty with Prevalence of Inadequate Nutrient Intake in Community-Dwelling Japanese Elderly Women: A Cross-Sectional Study. Asia Pac. J. Clin. Nutr..

[18] Stroup D.F., Berlin J.A., Morton S.C., Olkin I., Williamson G.D., Rennie D., Moher D., Becker B.J., Sipe T.A., Thacker S.B. (2000). Meta-Analysis of Observational Studies in Epidemiology: A Proposal for Reporting. Meta-Analysis Of Observational Studies in Epidemiology (MOOSE) Group. JAMA.

[19] Green S., Higgins J. (2005). Cochrane Handbook for Systematic Reviews of Interventions.

[20] Study Quality Assessment Tools|NHLBI, NIH. https://www.nhlbi.nih.gov/health-topics/study-quality-assessment-tools.

[21] Luo D., Wan X., Liu J., Tong T. (2018). Optimally Estimating the Sample Mean from the Sample Size, Median, Mid-Range, and/or Mid-Quartile Range. Stat. Methods Med. Res..

[22] Shi J., Luo D., Weng H., Zeng X.-T., Lin L., Chu H., Tong T. (2020). Optimally Estimating the Sample Standard Deviation from the Five-Number Summary. Res. Synth. Methods.

[23] Bartali B., Frongillo E.A., Bandinelli S., Lauretani F., Semba R.D., Fried L.P., Ferrucci L. (2006). Low Nutrient Intake Is an Essential Component of Frailty in Older Persons. J. Gerontol. Ser. A.

[24] Bollwein J., Diekmann R., Kaiser M.J., Bauer J.M., Uter W., Sieber C.C., Volkert D. (2013). Distribution but Not Amount of Protein Intake Is Associated with Frailty: A Cross-Sectional Investigation in the Region of Nürnberg. Nutr. J..

[25] Castaneda-Gameros D., Redwood S., Thompson J.L. (2017). Low Nutrient Intake and Frailty Among Overweight and Obese Migrant Women From Ethnically Diverse Backgrounds Ages 60 Years and Older: A Mixed-Methods Study. J. Nutr. Educ. Behav..

[26] Kobayashi S., Suga H., Sasaki S. (2017). Diet with a Combination of High Protein and High Total Antioxidant Capacity Is Strongly Associated with Low Prevalence of Frailty among Old Japanese Women: A Multicenter Cross-Sectional Study. Nutr. J..

[27] Wu S.Y., Yeh N.H., Chang H.Y., Wang C.F., Hung S.Y., Wu S.J., Pan W.H. (2021). Adequate Protein Intake in Older Adults in the Context of Frailty: Cross-Sectional Results of the Nutrition and Health Survey in Taiwan 2014–2017. Am. J. Clin. Nutr..

[28] Tamaki K., Kusunoki H., Tsuji S., Wada Y., Nagai K., Itoh M., Sano K., Amano M., Maeda H., Hasegawa Y. (2018). The Relationship between Dietary Habits and Frailty in Rural Japanese Community-Dwelling Older Adults: Cross-Sectional Observation Study Using a Brief Self-Administered Dietary History Questionnaire. Nutrients.

[29] Smit E., Winters-Stone K.M., Loprinzi P.D., Tang A.M., Crespo C.J. (2013). Lower Nutritional Status and Higher Food Insufficiency in Frail Older US Adults. Br. J. Nutr..

[30] Fried L.P., Tangen C.M., Walston J., Newman A.B., Hirsch C., Gottdiener J., Seeman T., Tracy R., Kop W.J., Burke G. (2001). Frailty in Older Adults: Evidence for a Phenotype. J. Gerontol. Ser. A.

[31] Sewo Sampaio P.Y., Sampaio R.A.C., Yamada M., Arai H. (2016). Systematic Review of the Kihon Checklist: Is It a Reliable Assessment of Frailty?. Geriatr. Gerontol. Int..

[32] Morley J.E., Malmstrom T.K., Miller D.K. (2012). A Simple Frailty Questionnaire (FRAIL) Predicts Outcomes in Middle Aged African Americans. J. Nutr. Health Aging.

[33] Ensrud K.E., Ewing S.K., Taylor B.C., Fink H.A., Cawthon P.M., Stone K.L., Hillier T.A., Cauley J.A., Hochberg M.C., Rodondi N. (2008). Comparison of 2 Frailty Indexes for Prediction of Falls, Disability, Fractures, and Death in Older Women. Arch. Intern. Med..

[34] Beasley J.M., Lacroix A.Z., Neuhouser M.L., Huang Y., Tinker L., Woods N., Michael Y., Curb J.D., Prentice R.L. (2010). Protein Intake and Incident Frailty in the Women’s Health Initiative Observational Study. J. Am. Geriatr. Soc..

[35] Huang C.H., Martins B.A., Okada K., Matsushita E., Uno C., Satake S., Kuzuya M. (2021). A 3-Year Prospective Cohort Study of -Dietary Patterns and Frailty Risk among Community-Dwelling Older Adults. Clin. Nutr..

[36] Otsuka R., Tange C., Tomida M., Nishita Y., Kato Y., Yuki A., Ando F., Shimokata H., Arai H. (2019). Dietary Factors Associated with the Development of Physical Frailty in Community-Dwelling Older Adults. J. Nutr. Health Aging.

[37] Sandoval-Insausti H., Perez-Tasigchana R.F., Lopez-Garcia E., Garcia-Esquinas E., Rodriguez-Artalejo F., Guallar-Castillon P. (2016). Macronutrients Intake and Incident Frailty in Older Adults: A Prospective Cohort Study. J. Gerontol. Ser. A.

[38] Shikany J.M., Barrett-Connor E., Ensrud K.E., Cawthon P.M., Lewis C.E., Dam T.T.L., Shannon J., Redden D.T. (2014). Macronutrients, Diet Quality, and Frailty in Older Men. J. Gerontol. Ser. A.

[39] Deer R.R., Volpi E. Protein Intake and Muscle Function in Older Adults. https://www.ncbi.nlm.nih.gov/pmc/articles/PMC4394186/.

[40] Marzetti E., Calvani R., Tosato M., Cesari M., Di Bari M., Cherubini A., Collamati A., D’Angelo E., Pahor M., Bernabei R. (2017). Sarcopenia: An Overview. Aging Clin. Exp. Res..

[41] Cruz-Jentoft A.J., Sayer A.A. (2019). Sarcopenia. Lancet.

[42] Picca A., Coelho-Junior H.J., Calvani R., Marzetti E., Vetrano D.L. (2022). Biomarkers Shared by Frailty and Sarcopenia in Older Adults: A Systematic Review and Meta-Analysis. Ageing Res. Rev..

[43] Landi F., Calvani R., Tosato M., Martone A.M., Ortolani E., Savera G., D’Angelo E., Sisto A., Marzetti E. (2016). Protein Intake and Muscle Health in Old Age: From Biological Plausibility to Clinical Evidence. Nutrients.

[44] Morley J.E., von Haehling S., Anker S.D., Vellas B. (2014). From Sarcopenia to Frailty: A Road Less Traveled. J. Cachexia Sarcopenia Muscle.

[45] Atherton P.J., Etheridge T., Watt P.W., Wilkinson D., Selby A., Rankin D., Smith K., Rennie M.J. (2010). Muscle Full Effect after Oral Protein: Time-Dependent Concordance and Discordance between Human Muscle Protein Synthesis and MTORC1 Signaling. Am. J. Clin. Nutr..

[46] Bohé J., Low A., Wolfe R.R., Rennie M.J. (2003). Human Muscle Protein Synthesis Is Modulated by Extracellular, Not Intramuscular Amino Acid Availability: A Dose-Response Study. J. Physiol..

[47] Tang J.E., Moore D.R., Kujbida G.W., Tarnopolsky M.A., Phillips S.M. (2009). Ingestion of Whey Hydrolysate, Casein, or Soy Protein Isolate: Effects on Mixed Muscle Protein Synthesis at Rest and Following Resistance Exercise in Young Men. J. Appl. Physiol..

[48] Greenhaff P.L., Karagounis L.G., Peirce N., Simpson E.J., Hazell M., Layfield R., Wackerhage H., Smith K., Atherton P., Selby A. (2008). Disassociation between the Effects of Amino Acids and Insulin on Signaling, Ubiquitin Ligases, and Protein Turnover in Human Muscle. Am. J. Physiol. Endocrinol. Metab..

[49] Wilkinson D.J., Hossain T., Hill D.S., Phillips B.E., Crossland H., Williams J., Loughna P., Churchward-Venne T.A., Breen L., Phillips S.M. (2013). Effects of Leucine and Its Metabolite β-Hydroxy-β-Methylbutyrate on Human Skeletal Muscle Protein Metabolism. J. Physiol..

[50] Wilkinson D.J., Piasecki M., Atherton P.J. (2018). The Age-Related Loss of Skeletal Muscle Mass and Function: Measurement and Physiology of Muscle Fibre Atrophy and Muscle Fibre Loss in Humans. Ageing Res. Rev..

[51] Lexell J., Taylor C.C., Sjöström M. (1988). What is the cause of the ageing atrophy?: Total number, size and proportion of different fiber types studied in whole vastus lateralis muscle from 15- to 83-year-old men. J. Neurol. Sci..

[52] Nilwik R., Snijders T., Leenders M., Groen B.B.L., van Kranenburg J., Verdijk L.B., Van Loon L.J.C. (2013). The Decline in Skeletal Muscle Mass with Aging Is Mainly Attributed to a Reduction in Type II Muscle Fiber Size. Exp. Gerontol..

[53] Scott W., Stevens J., Binder-Macleod S. (2001). Human Skeletal Muscle Fiber Type Classifications. Phys. Ther..

[54] van Vliet S., Burd N.A., van Loon L.J. (2015). The Skeletal Muscle Anabolic Response to Plant- versus Animal-Based Protein Consumption. J. Nutr..

[55] Gorissen S.H.M., Witard O.C. (2018). Characterising the Muscle Anabolic Potential of Dairy, Meat and Plant-Based Protein Sources in Older Adults. Proc. Nutr. Soc..

[56] Li C.-Y., Fang A.-P., Ma W.-J., Wu S.-L., Li C.-L., Chen Y.-M., Zhu H.-L. (2019). Amount Rather than Animal vs Plant Protein Intake Is Associated with Skeletal Muscle Mass in Community-Dwelling Middle-Aged and Older Chinese Adults: Results from the Guangzhou Nutrition and Health Study. J. Acad. Nutr. Diet..

[57] Borack M.S., Volpi E. (2016). Efficacy and Safety of Leucine Supplementation in the Elderly. J. Nutr..

[58] Bolster D.R., Vary T.C., Kimball S.R., Jefferson L.S. (2004). Leucine Regulates Translation Initiation in Rat Skeletal Muscle Via Enhanced EIF4G Phosphorylation. J. Nutr..

[59] Dardevet D., Sornet C., Balage M., Grizard J. (2000). Stimulation of in Vitro Rat Muscle Protein Synthesis by Leucine Decreases with Age. J. Nutr..

[60] Coelho-Junior H.J., Calvani R., Gonçalves I.O., Rodrigues B., Picca A., Landi F., Bernabei R., Uchida M.C., Marzetti E. (2019). High Relative Consumption of Vegetable Protein Is Associated with Faster Walking Speed in Well-Functioning Older Adults. Aging Clin. Exp. Res..

[61] Schoufour J.D., Franco O.H., Kiefte-De Jong J.C., Trajanoska K., Stricker B., Brusselle G., Rivadeneira F., Lahousse L., Voortman T. (2019). The Association between Dietary Protein Intake, Energy Intake and Physical Frailty: Results from the Rotterdam Study. Br. J. Nutr..

[62] Ten Haaf D.S., Van Dongen E.J., Nuijten M.A., Eijsvogels T.M., De Groot L.C., Hopman M.T. (2018). Protein Intake and Distribution in Relation to Physical Functioning and Quality of Life in Community-Dwelling Elderly People: Acknowledging the Role of Physical Activity. Nutrients.

[63] Loenneke J.P., Loprinzi P.D., Murphy C.H., Phillips S.M. (2016). Per Meal Dose and Frequency of Protein Consumption Is Associated with Lean Mass and Muscle Performance. Clin. Nutr..

[64] Moore D.R., Churchward-Venne T.A., Witard O., Breen L., Burd N.A., Tipton K.D., Phillips S.M. (2015). Protein Ingestion to Stimulate Myofibrillar Protein Synthesis Requires Greater Relative Protein Intakes in Healthy Older versus Younger Men. J. Gerontol. Ser. A.

[65] Watanabe Y., Hirano H., Arai H., Morishita S., Ohara Y., Edahiro A., Murakami M., Shimada H., Kikutani T., Suzuki T. (2017). Relationship Between Frailty and Oral Function in Community-Dwelling Elderly Adults. J. Am. Geriatr. Soc..

[66] Landi F., Calvani R., Tosato M., Martone A.M., Ortolani E., Savera G., Sisto A., Marzetti E. (2016). Anorexia of Aging: Risk Factors, Consequences, and Potential Treatments. Nutrients.

[67] Bernabei R., Landi F., Calvani R., Cesari M., Del Signore S., Anker S.D., Bejuit R., Bordes P., Cherubini A., Cruz-Jentoft A.J. (2022). Multicomponent Intervention to Prevent Mobility Disability in Frail Older Adults: Randomised Controlled Trial (SPRINTT Project). BMJ.

