# Supplementary material for: Protein Intake and Frailty in Older Adults: A Systematic Review and Meta-Analysis of Observational Studies
Source: Nutrients. 2022 Jul 5;14(13):2767. doi: 10.3390/nu14132767 (PMC9269106; doi:10.3390/nu14132767)
Supplement: Supplementary file 1 [file nutrients-14-02767-s001.zip › nutrients-1799711-supplementary.pdf]

**Supplementary Material S1. The complete search strategy.**

**PUBMED**

1. Protein intake AND frailty [MESH];
2. Protein intake AND frailty index;
3. Protein intake AND fragility;
4. Protein intake AND Frailties;
5. Protein intake AND Frailness;
6. Protein intake AND Frailty Syndrome;
7. Protein intake AND Debility;
8. Protein intake AND Debilities;
9. Protein intake AND Frail Elderly;
10. Protein intake AND Physical Frailty;
11. Protein intake AND Clinical Frailty Scale;
12. Protein intake AND FRAIL Scale;
13. Protein intake AND Frailty Phenotype;
14. Protein intake AND Deficit Accumulation Index;
15. Protein intake AND Cognitive Frailty;
16. Protein consumption AND frailty [MESH];
17. Protein consumption AND frailty index;
18. Protein consumption AND fragility;
19. Protein consumption AND Frailties;
20. Protein consumption AND Frailness;
21. Protein consumption AND Frailty Syndrome;
22. Protein consumption AND Debility;
23. Protein consumption AND Debilities;
24. Protein consumption AND Frail Elderly;
25. Protein consumption AND Physical Frailty;
26. Protein consumption AND Clinical Frailty Scale;
27. Protein consumption AND FRAIL Scale;
28. Protein consumption AND Frailty Phenotype;
29. Protein consumption AND Deficit Accumulation Index;
30. Protein consumption AND Cognitive Frailty;

**EMBASE***

1. Protein intake AND Frailty AND Aged;
2. Protein intake AND Frailty AND Older Adults;
3. Protein intake AND Frailty AND Elderly;

*All searchers were conducted using the filter *Title, Abstract, Author Keywords*

**<u>SCOPUS*</u>**

1. Protein intake AND Frailty AND Aged;
2. Protein intake AND Frailty AND Older Adults;
3. Protein intake AND Frailty AND Elderly;
4. Protein consumption AND Frailty AND Aged;
5. Protein consumption AND Frailty AND Older Adults;
6. Protein consumption AND Frailty AND Elderly;

*All searchers were conducted using the filter *Article title, Abstract, Keywords*

**<u>EBSCO (AgeLine, CINAHL, Food Science Source)</u>**

1. Protein intake AND Frailty AND Aged;
2. Protein intake AND Frailty AND Older Adults;
3. Protein intake AND Frailty AND Elderly;
4. Protein intake AND Frailty AND Senior;
5. Protein intake AND Frailty AND Geriatrics;
6. Protein consumption AND Frailty AND Aged;
7. Protein consumption AND Frailty AND Older Adults;
8. Protein consumption AND Frailty AND Elderly;
9. Protein consumption AND Frailty AND Senior;
10. Protein consumption AND Frailty AND Geriatrics;

**Supplementary Material S2. Six articles which were excluded.**

AGE

1. Schoufour, J. D., Franco, O. H., Kiefte-de Jong, J. C., Trajanoska, K., Stricker, B., Brusselle, G., ... & Voortman, T. (2019). The association between dietary protein intake, energy intake and physical frailty: results from the Rotterdam Study. British Journal of Nutrition, 121(4), 393-401.
2. Verspoor, E., Voortman, T., van Rooij, F. J., Rivadeneira, F., Franco, O. H., Kiefte-de Jong, J. C., & Schoufour, J. D. (2020). Macronutrient intake and frailty: the Rotterdam Study. European journal of nutrition, 59(7), 2919-2928.
3. Villani, A., Barrett, M., McClure, R., & Wright, H. (2021). Protein intake is not associated with functional biomarkers of physical frailty: A cross-sectional analysis of community-dwelling older adults with type 2 diabetes mellitus. Nutrition, Metabolism and Cardiovascular Diseases, 31(3), 827-833.

DID NOT ASSESS PROTEIN INTAKE

4. Huang, C. H., Martins, B. A., Okada, K., Matsushita, E., Uno, C., Satake, S., & Kuzuya, M. (2021). A 3-year prospective cohort study of dietary patterns and frailty risk among community-dwelling older adults. Clinical Nutrition, 40(1), 229-236.

5. Lana, A., Rodriguez-Artalejo, F., & Lopez-Garcia, E. (2015). Dairy consumption and risk of frailty in older adults: a prospective cohort study. Journal of the American Geriatrics Society, 63(9), 1852-1860.
6. Yamaguchi, M., Yamada, Y., Nanri, H., Nozawa, Y., Itoi, A., Yoshimura, E., ... & Kyoto-Kameoka Study Group. (2018). Association between the frequency of protein-rich food intakes and Kihon-Checklist frailty indices in older Japanese adults: the Kyoto-Kameoka study. Nutrients, 10(1), 84.

**Supplementary Material S3**

**Supplementary Material S3**. Quality analysis

| Authors | 1 | 2 | 3 | 4 | 5 | 6 | 7 | 8 | 9 | 10 | 11 | 12 | 13 | 14 | Overall score (0/11) |
|---|---|---|---|---|---|---|---|---|---|---|---|---|---|---|---|
| *Cross-sectional* | | | | | | | | | | | | | | | |
| Bartali et al. | Y | Y | Y | Y | NR | NA | NA | N | Y | N | Y | NR | NA | Y | 7 |
| Bollwein et al. | Y | Y | Y | Y | NR | NA | NA | N | Y | N | Y | NR | NA | Y | 7 |
| Castaneda-Gameros et al. | Y | Y | NR | Y | NR | NA | NA | N | Y | N | Y | NR | NA | Y | 6 |
| Coelho-Junior et al. | Y | Y | Y | Y | NR | NA | NA | Y | Y | N | Y | N | NA | N | 7 |
| Hayashi et al. | Y | Y | Y | Y | NR | NA | NA | Y | Y | N | Y | NR | NA | Y | 8 |
| Kaimoto et al. | Y | Y | Y | Y | NR | NA | NA | Y | Y | N | Y | NR | NA | Y | 8 |
| Kobayashi et al. | Y | Y | Y | Y | NR | NA | NA | Y | Y | N | Y | NR | NA | Y | 8 |
| Kobayashi et al. | Y | Y | Y | Y | NR | NA | NA | Y | Y | N | Y | NR | NA | Y | 8 |
| Rahi et al. | Y | Y | Y | Y | NR | NA | NA | N | Y | N | Y | NR | NA | Y | 7 |
| Smit et al. | Y | Y | Y | Y | NR | NA | NA | Y | Y | N | Y | NR | NA | Y | 8 |
| Tamaki et al. | Y | Y | Y | Y | NR | NA | NA | N | Y | N | Y | NR | NA | N | 6 |
| Wu et al. | Y | Y | Y | Y | NR | NA | NA | N | Y | N | Y | NR | NA | Y | 7 |
| *Longitudinal* | | | | | | | | | | | | | | | |
| Beasley et al. | Y | Y | Y | Y | NR | Y | Y | Y | Y | N | Y | NR | NR | Y | 10 |
| Huang et al. | Y | Y | Y | Y | NR | Y | Y | N | Y | N | Y | NR | Y | Y | 10 |
| Otsuka et al. | Y | Y | Y | Y | NR | Y | Y | N | Y | N | Y | NR | NR | Y | 9 |
| Sandoval-Insausti et al. | Y | Y | Y | Y | NR | Y | Y | Y | Y | N | Y | NR | NR | Y | 10 |
| Shikany et al. | Y | Y | NR | Y | NR | Y | Y | N | Y | N | Y | NR | NR | Y | 8 |
